# Evaluation of Different Bearing Fault Classifiers in Utilizing CNN Feature Extraction Ability

**DOI:** 10.3390/s22093314

**Published:** 2022-04-26

**Authors:** Wenlang Xie, Zhixiong Li, Yang Xu, Paolo Gardoni, Weihua Li

**Affiliations:** 1 School of Mechanical, Materials, Mechatronic and Biomedical Engineering, University of Wollongong, Wollongong, NSW 2522, Australia; wx528@uowmail.edu.au; 2 Faculty of Mechanical Engineering, Opole University of Technology, 45-758 Opole, Poland; z.li@po.edu.pl; 3 School of Engineering, Ocean University of China, Qingdao 266110, China; xu499345@163.com; 4 Department of Civil and Environmental Engineering, University of Illinois at Urbana-Champaign, Champaign, IL 61820, USA; gardoni@illinois.edu

**Keywords:** bearing fault diagnosis, deep learning, machine learning, convolutional neural network, feature extraction, bearing fault classifier

## Abstract

In aerospace, marine, and other heavy industries, bearing fault diagnosis has been an essential part of improving machine life, reducing economic losses, and avoiding safety problems caused by machine bearing failures. Most existing bearing fault diagnosis methods face challenges in extracting the fault features from raw bearing fault data. Compared with traditional methods for bearing fault characteristics extraction, deep neural networks can automatically extract intrinsic features without expert knowledge. The convolutional neural network (CNN) was utilized most widely in extracting representative features of bearing faults. Fundamental to this, the hybrid models based on the CNN and individual classifiers were proposed to diagnose bearing faults. However, CNN may not be suitable for all bearing fault classifiers. It is crucial to identify the classifiers which can maximize the CNN feature extraction ability. In this paper, four hybrid models based on CNN were built, and their fault detection accuracy and efficiency were compared. The comparative analysis showed that the random forest (RF) and support vector machine (SVM) could make full use of the CNN feature extraction ability.

## 1. Introduction

Bearing fault diagnosis has been developed sustainably for years to avoid massive economic loss and unpredictable safety accidents caused by bearing faults [1]. Most diagnosis methods face challenges in extracting critical features from raw bearing fault data. Traditional feature extraction methods are fundamental to vibration signals generated by the contacts of defective and normal elements [2]. However, these classical methods have difficulty explaining bearing fault conditions and detection accuracy. To make progress in this situation, feature extraction based on machine learning/deep learning algorithms was utilized in bearing fault diagnosis [3]. The core principle of this artificial intelligence (AI)-based feature extraction is to find critical features in the raw data automatically; that means no human factors such as professional knowledge are needed in the feature extraction process [4].

Deep learning is currently the most popular feature extraction technique for machine defect detection and was proven to automatically extract the critical fault characteristics. Typical deep learning algorithms include the denoising auto-encoders (DAE) [5], the deep belief network (DBN) [6], and the sparse auto-encoders (SAE) [7], which are able to extract one-dimensional linear information from the raw bearing vibration signal. Meanwhile, the restricted Boltzmann machine (RBM) [8] and the deep Boltzmann machine (DBM) [9] can solve the information-missing problem that exists in the bearing vibration data, while the recurrent neural network (RNN) [10] and the convolutional neural network (CNN) [11] have shown great potential in multi-dimensional analysis for the bearing vibration data. The CNN is the most incentivized bearing fault diagnosis method [12]. This approach does not require introducing an unsupervised pre-training process and prior expert knowledge to train the detection model and has the qualified capability to eliminate the noise that exists in the fault datasets [13]. More importantly, compared with other deep learning models, CNN has outstanding ability in multi-dimensional image feature extraction, especially in two-dimensional (2D) image extraction [14]. The feature extraction ability of the CNN has inspired researchers to propose hybrid bearing fault diagnosis models based on the CNN and classical classifiers, where the CNN is pre-trained to extract the bearing fault features to feed the classifiers. The hybrid CNN-gcForest model [15], CNN-RF model [16], and CNN-SVM model [17] were developed for bearing fault diagnosis. Apart from these hybrid models, many other classifiers can be combined with a pre-trained CNN model. In existing individual bearing fault classifiers, the back propagation (BP) method was proposed to classify the bearing fault by the actual executed number of times of the model [18]. Moreover, the radial basis function (RBF) is another effective classifier method utilized in the bearing fault diagnosis, which has the potential to reduce the effect of noise and achieve good robustness [19]. The long short-term memory (LSTM), a variant of the RBF model, was proven suitable to combine with other deep learning models. For example, a collaborative CNN-LSTM hybrid model was proposed to detect COVID-19 from X-rays image data [20]. However, the CNN may not work out with every classifier. It is crucial to determine the most suitable classifiers to maximize the feature extraction ability of the CNN.

In this paper, four preliminary hybrid CNN models are examined. The bearing features extracted from the CNN model are fed into the gcForest, random forest, SVM, and LSTM classifiers. The comparative analysis between these hybrid models was carried out using the bearing vibration data. The contributions of this paper are: (i) evaluating the CNN feature extraction ability under different sizes of dataset; (ii) evaluating different classifiers in making use of CNN feature extraction ability in diagnosis accuracy; (iii) evaluating different classifiers in making use of CNN feature extraction ability in diagnosis.

## 2. The Proposed Method

**Figure 1:**
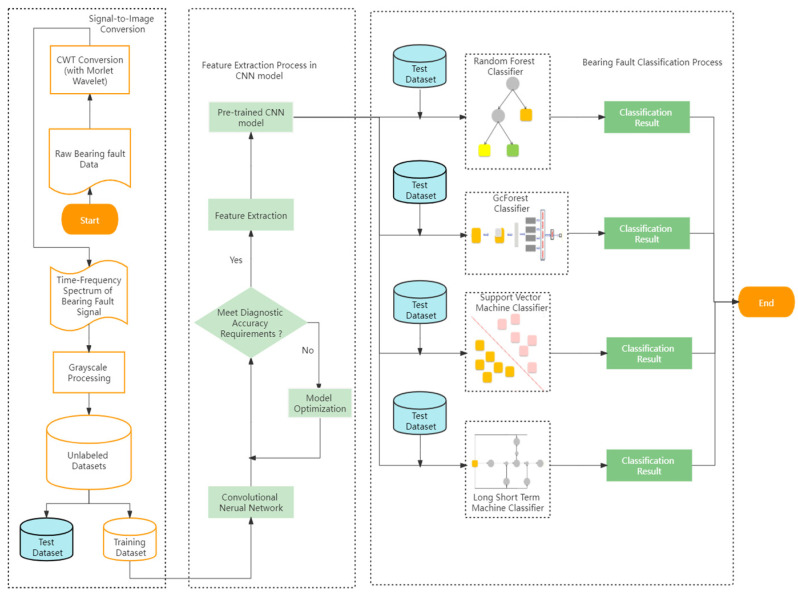
The workflow of the proposed method.

The hybrid CNN model consists of three parts: one single-to-image conversion part, one feature extraction part, and one bearing fault classification part, as shown in Figure 1.

In the signal-to-image conversion part, the overlap method was utilized here to truncate the one-dimensional plain bearing fault signal from the Case Western Reserve University (CWRU) bearing fault datasets [21]. The truncated data points were converted into the continuous wavelet transform (CWT) images [22]. In the continuous wavelet family, the Morlet wavelet was selected because it is the most reasonable wavelet to describe the bearing fault features in the time-frequency domain [23].

(1) $$\psi \left(t\right)=exp^{-\frac{t^{2}}{2}}cos\left(5t\right)\;$$

To eliminate the negative impact of the color information on the diagnosis accuracy, the original CWT images were converted to grayscale images. All the images were stored in the unlabeled datasets, classified according to the fault location principle.

**Figure 2:**
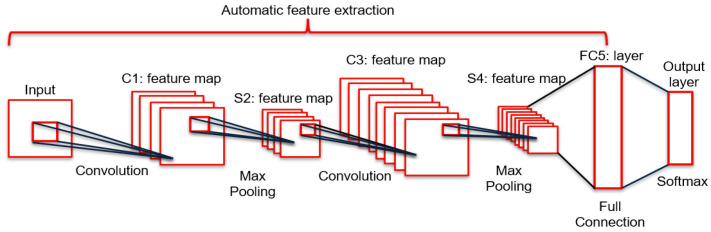
The architecture of constructed CNN model.

The feature extraction part utilized the CNN to extract the bearing fault features from the grayscale images. In Figure 2, apart from the input layer, the architecture of the CNN model in this paper included two convolutional layers, two pooling layers, and one full connection layer.

First, according to Equation (2), in the convolutional neural network, the input fault image was divided into several sections, and the feature of each section was represented as input $x_{i}$.The preliminary fault characteristic consists of two components. One is the convolution of the weight $w_{i}$ and input $x_{i}$, the other is the bias b. Then, the pooling operation will be achieved to reduce the dimension of the fault characteristic initially extracted in the convolutional layer.

(2) $$y=\sum_{i}w_{i}x_{i}+b\;$$

Moreover, the fault features were mapped to a sample label space to eliminate the impact from feature locations, and the features were classified in the full connection (FC5) layer. Generally, the detailed classification results were outputted in the output layer. Then, as shown in Figure 2, the classification results are measured in terms of whether they meet the requirements for diagnostic accuracy. If not, the parameters in the CNN model will be optimized. Then, the CNN model, which had the highest average diagnosis accuracy, was saved as the pre-trained CNN model.

The fault classification part replaced the CNN output layer with four popular classifiers, e.g., RF, SVM, gcForest, and LSTM.

First, in the CNN-RF hybrid model, which is illustrated in Figure 1, the bearing fault feature extracted by the full connection layer of the pre-trained CNN model was reshaped to be able to feed into the random forest classifier. According to the input data, the corresponding number of decision trees will be constructed, and each decision tree will produce one classification result. Furthermore, the result will be determined by a voting mechanism based on each decision tree’s output.

Then, in the CNN-gcForest hybrid model, the bearing fault diagnosis mechanism was briefly similar to that of the CNN-RF hybrid model. According to Zhou [24], the gcForest model includes two components of multi-grain scanning and cascade forest. After reshaping the features extracted from the CNN model and feeding them to the gcForest, in the multi-grained scanning process, a *K* × *K* dimensional slide window was utilized in obtaining the training samples from the raw input data while the size of the raw input data is *N* × *N* and the slide step is *H*.

(3) $$J={\left[\left(N-K\right)/H+1\right]}^{2}\;$$

The amount of the training samples is *J*, which was determined by the raw input data, slide window, and slide step. Moreover, the features extracted from the training samples will be fed into the cascade forest. In the cascade forest, the average probability distribution of bearing fault was utilized in generating the classification results.

Furthermore, in the CNN-SVM hybrid model, as shown in Figure 1, kernel tricks were utilized in solving bearing fault multi-classification tasks. With the help of kernel tricks, in the CNN-SVM hybrid model, *N* classification task was separated into *N* binary task. Furthermore, the final classification result could be obtained.

In addition, in the CNN-LSTM hybrid model, one LSTM layer was added between the pooling and the full connection layers. The fault features extracted from the CNN model will be fed into the LSTM layer to learn the relationships among bearing fault data and the time series. The final classification result can be obtained after identifying the time-series relationship of the different types of bearing fault data.

## 3. Results and Analysis

The diagnosis accuracy and fault classification time were regarded as the model performance evaluation principles to evaluate four hybrid models. The accuracy and computing time of the hybrid models were comparatively analyzed. The confusion matrix was utilized to measure the model performance in each type of bearing fault.

The PyCharm 2020 ×64 was utilized in the model experiments. The computer configurations were: 2.60 GHz CPU, 64-bit operating system, 1 TB HDD, 256 GB SSD, 16 GB RMB, and Inter (R) UHD Graphics 630 NVIDIA GeForce RTX 2060.

In this study, the bearing fault datasets provided by the CWRU Bearing Data Center were utilized to evaluate the classifier’s suitability for the pre-trained CNN model. The datasets included the raw bearing fault signals under four different working loads, which were 0 horsepower load, 1 horsepower, 2 horsepower load, and 3 horsepower. The principle underlying dividing the fault signal was bearing fault location. In the CWRU dataset, the faults were divided into the inner race fault (IR), outer race fault (OR), ball fault (B), and health bearing data (HD). Specifically, the data were further divided into three types of faults according to the fault diameter, which was 0.007 inches, 0.014 inches, and 0.021 inches. This study included one small-scale dataset (200 training datasets/100 test datasets), under 2 horsepower working loads, and one large-scale dataset (600 training/300 test datasets), which was the mixture of datasets under 1–3 horsepower working loads. The details of dataset division are shown in Table 1.

In the training process, prior to feeding the raw bearing fault signal to the CNN model, the overlap approach was first utilized in the signal-to-image conversion stage by putting 1024 sample data points in one image to obtain more effective datasets. After intercepting the first image, the interception window was panned to the right by 384 data points. The second and first images had 860 data points overlapping, allowing for more images and allowing each image to have common features.

**Figure 3:**
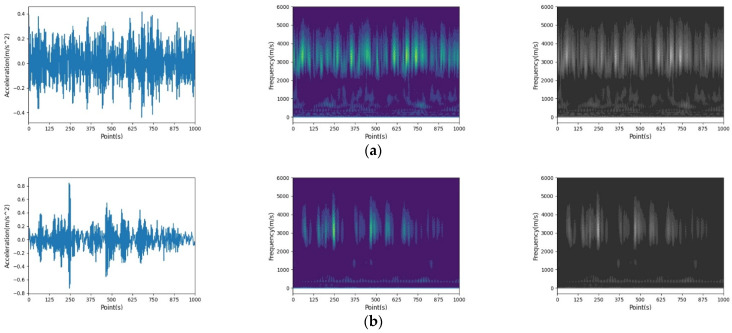

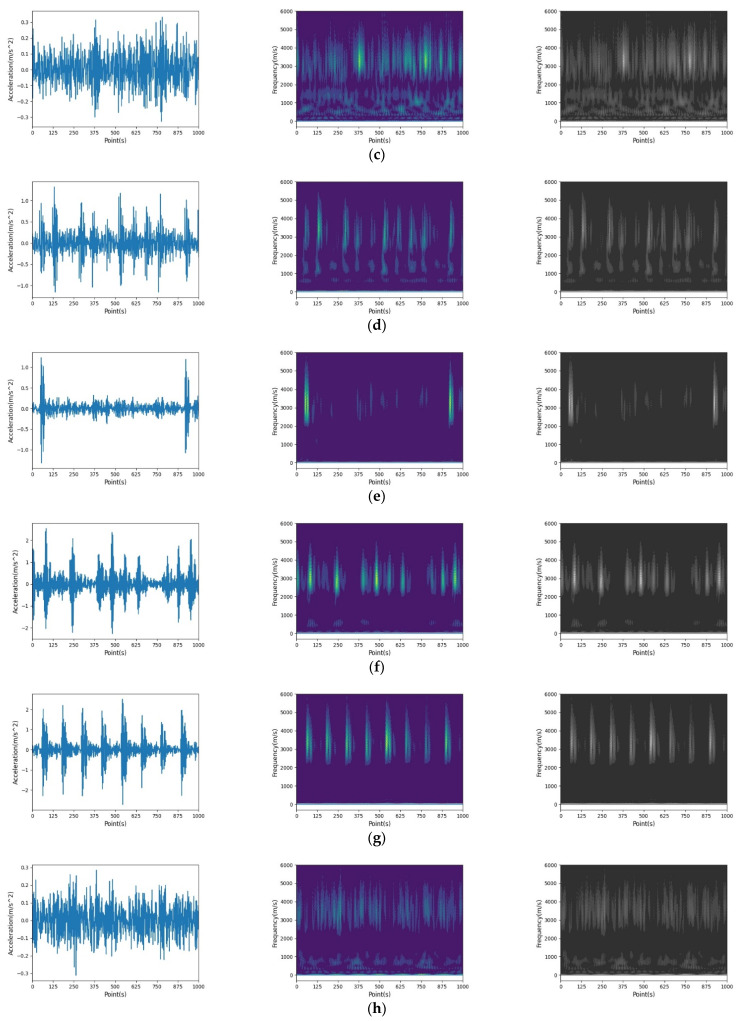

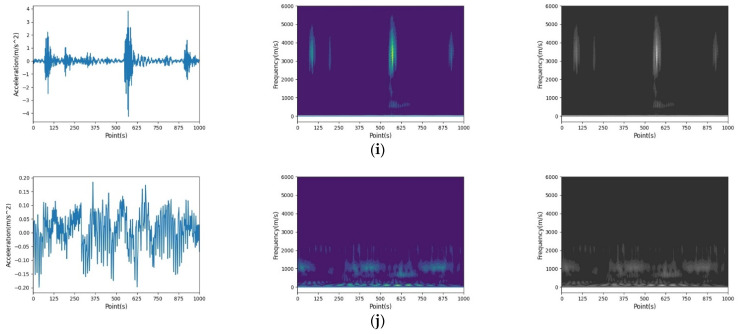
The raw bearing fault waveform, CWT images, and grayscale images of: (**a**) B_07, (**b**) B_014, (**c**) B_021, (**d**) IR_007, (**e**) IR_014, (**f**) IR_021, (**g**) IR_007, (**h**) OR_014, (**i**) OR_021, and (**j**) HD.

Then, the CWT method was utilized in processing the raw bearing fault waveform. The CWT images were converted to grayscale images to eliminate the impact from disturbing color information. The details of raw bearing fault signal in time-frequency spectrum, CWT images, and grayscale images of different types of bearing fault are shown in Figure 3. In Figure 3, the waveform of each type of defective bearing was different from the wavelet of bearing in health condition (the image labeled Normal_Baseline), which means that the images processed by the CWT method have the capability of been used in training and to test the proposed model.

**Figure 4:**
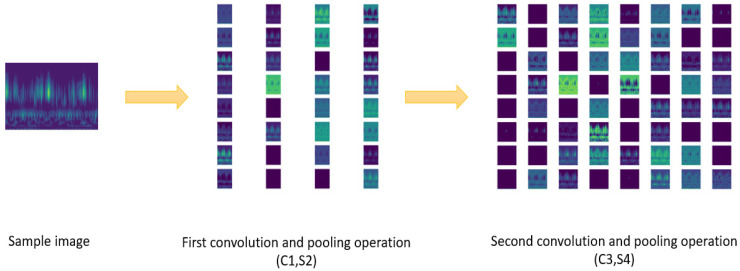
The convolutional and pooling operations of the CNN component in the proposed method.

After processing the raw bearing fault signal, the grayscale images were fed into the CNN model to extract the critical feature. Figure 4 illustrates the mechanism of the CNN feature extraction operation by visualizing the feature map in the convolutional and max-pooling layers.

For the CNN model utilized in this paper, the shallow layers represented the first convolution layer (C1) and first max-pooling layer (S2), while the deep layers represented the second convolution layer (C3) and second max-pooling layer (S4). Different kernels in C1 and C3 determined the quantitative differences of feature maps.

**Figure 5:**
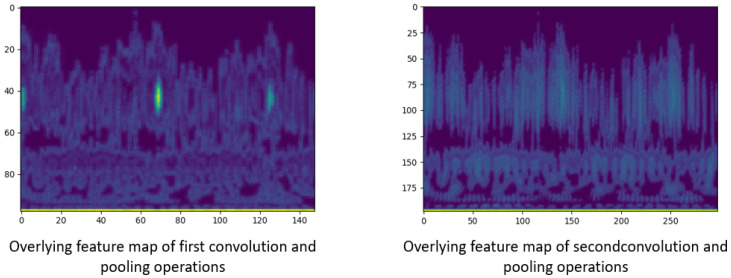
The overlaying feature maps of convolution and pooling operation.

Furthermore, via integrating the feature maps into one overlaying feature map, more mechanisms can be revealed. Figure 5 illustrates the overlaying feature maps of the first convolution and pooling operation (C1, S2) and the maps of the second convolution and pooling operation (C3, S4).

As can be seen in Figure 5, shallow layers extracted texture and detailed feature, while deep layers extracted contour and shape feature. Generally, shallow layers contained more features and had the ability to extract key features, and deep layers extracted representative features. In addition, as the depth of the network increased, the resolution of the image became smaller.

In the feature extraction process, the CNN model was supposed to meet principles to prove that the constructed CNN was qualified to extract crucial characteristics. These requirements were set before the construction of the formal model. The principles are shown below.

1. The classification accuracy of the CNN model in the training dataset and validation dataset needs to be up to 95%.
2. The training and validation loss of the CNN model needs to be reduced to a level close to 0.
3. The validation curve needs to fit the training curve.

**Figure 6:**
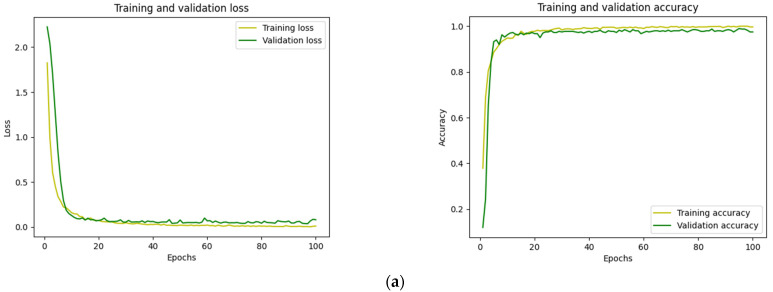

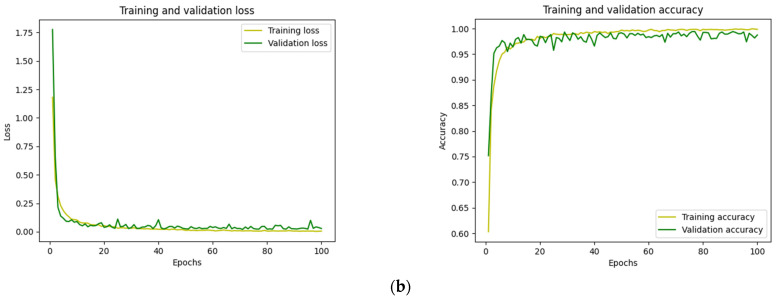
The training and validation loss curve and the training and validation accuracy of: (**a**) individual dataset and (**b**) hybrid dataset.

Figure 6 shows the training validation and their loss curves of the CNN.

In Figure 6, after 100 training epochs, the loss and accuracy curves met the principles mentioned above, indicating that the pre-trained CNN model was reasonable. For both datasets, the validation accuracy curves matched the training accuracy curves precisely, and both can achieve 97% and above accuracy.

The last component of the proposed method was the bearing fault classification component. The hyper-parameters of classifiers utilized in this study are illustrated in Table 2. Specifically, the hyper-parameters were determined by trial-to-error analysis and grid search strategy.

In this case, the model’s performance was evaluated regarding bearing diagnosis accuracy and bearing diagnosis efficiency. Specifically, the bearing diagnosis accuracy and the time taken to identify bearing faults for the test set were chosen as the basis for a comparative analysis of different classifiers and different hybrid models based on CNN models. To minimize errors and to validate the experimental model, the statistical bearing fault diagnosis accuracy and time were averaged over 20 replicate trials. The details are shown in Table 3.

For absolute values of diagnosis accuracy, gcForest was the individual model with the highest accuracy for small-scale datasets, and the LSTM model performed the worst. Among the hybrid models with improved classifiers based on the CNN models, the CNN-gcForest and CNN-RF models were the most accurate, reaching 99%, while the CNN-SVM and CNN-LSTM models were slightly less accurate. For large-scale datasets, the experimental results of the individual classifiers were similar. Among the hybrid models, CNN-SVM and CNN-gcForest were the most accurate. Regarding bearing diagnosis efficiency, the RF was the most efficient of all classifiers for both small and large datasets, and this result also applies to all hybrid models.

Specifically, the diagnosis accuracy evaluation method was shown in Equation (4). *D* represented the difference between hybrid model accuracy and the corresponding individual classifier accuracy in the same dataset, while $P_{1}$ represented CNN-classifier hybrid model average accuracy (%) and $\;P_{2}$ represented the individual classifier average accuracy (%).

A positive difference means that the diagnosis accuracy of the individual classifier was improved by the CNN feature extraction ability. The larger the difference, the better the effect of the CNN’s feature extraction function on the classifier, and the smaller the difference, the less the effect of the CNN’s feature extraction process on the classifier.

(4) $$D=P_{1}-P_{2}$$

(5) $$R=\frac{T_{1}}{T_{2}}$$

In terms of diagnosis time, the method to measure the diagnosis efficiency in the same dataset was shown in Equation (5). *R* represented the ratio between diagnosis time of the hybrid model and the individual classifier in the same dataset, while $T_{1}$ represented the hybrid model diagnosis time (s) and $\;T_{2}$ represented the individual classifier diagnosis time (s).

The comparative analysis results to evaluate the gcForest, RF, SVM, and LSTM in utilizing the CNN feature extraction ability were obtained. The details are illustrated in Table 4.

In Table 4, the difference (D) between the accuracy of CNN-SVM and the SVM was the largest in the small-scale dataset. The difference between accuracy of the CNN-RF and RF model in the large-scale dataset was the largest. This indicated that the feature extraction ability improved for all classifiers in the same dataset, while random forest (RF) and support vector machine (SVM) had the best-in-class performance, respectively, in collaborating with a pre-trained CNN model and utilized the CNN feature extraction ability to the maximum extent.

Moreover, in Table 4, the ratio among the diagnosis time of all hybrid models to individual models was less than 1 in the same dataset, with the ratio of the CNN-SVM hybrid model to the SVM model being close to 0.1, which is the smallest, and the ratio of the CNN-RF hybrid model to the RF classifier being the largest. The above results show that the CNN-model-based hybrid model approach improved diagnosis efficiency for different classifiers in the same dataset. Moreover, the results indicated that SVM utilized the CNN feature extraction ability to improve the diagnosis efficiency.

We further analyzed the hybrid model’s improvement of the classifier’s diagnosis accuracy for each bearing fault subclass. The confusion matrix was utilized to evaluate the diagnostic accuracy of the models in each type of bearing fault. The confusion matrix is intended to predict the prediction labels for the test data when the model performs recognition prediction on the test set, and when the predicted labels match the true labels, the model classifies accurately. In the confusion matrix, the model’s classification is not only shown by the percentage data but also by the set color intervals.

**Figure 7:**
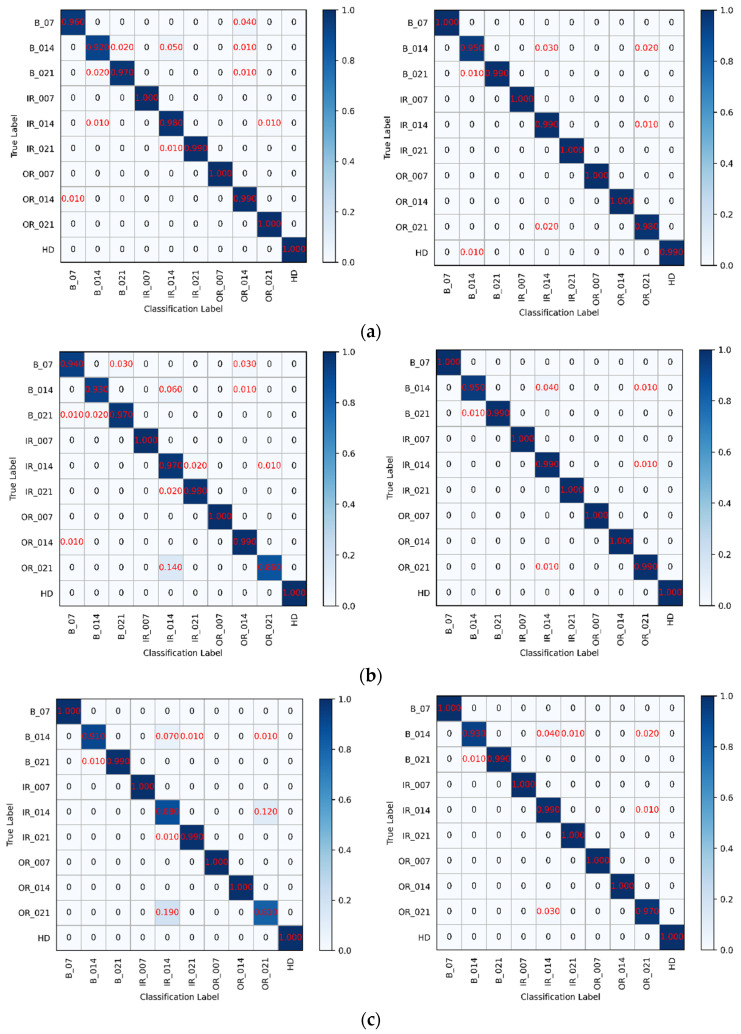

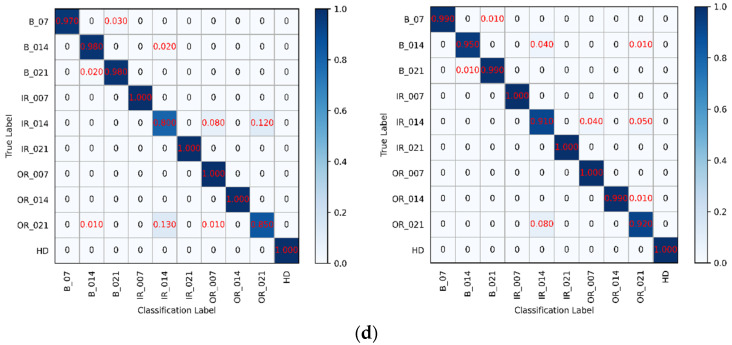
The sample confusion matrix of (**a**) gcForest and CNN-gcForest model; (**b**) RF and CNN-RF model; (**c**) SVM and CNN-SVM model; (**d**) LSTM and CNN-LSTM model.

In the confusion matrix shown in Figure 7, all of the diagnostic accuracy of each fault type in hybrid models is higher than 90%, while the diagnosis accuracy of certain fault types is lower than 85% in individual classifiers, which is not satisfactory. The individual RF model misclassified 14% 0.021-inch outer race bearing fault to 0.014-inch inner race bearing fault, while the CNN-RF hybrid model only misclassified 1%-inch outer race bearing fault to 0.014-inch inner race bearing fault. Furthermore, the individual SVM model misclassified 19% 0.021-inch outer race bearing fault to 0.014-inch inner race bearing fault and 12% 0.014-inch race bearing fault to 0.021-inch race bearing fault. Apart from these two classifiers, the CNN model did not have good performance in improving the diagnosis accuracy of classifiers in each type of fault. These indicated that RF and SVM have the potential to make full use of CNN feature extraction ability in improving the diagnosis accuracy in each subclass of the bearing faults.

In terms of diagnostic accuracy and efficiency, the CNN model had less significant improvement for gcForest and no significant advantage in diagnosis efficiency. The LSTM model performed the worst among the four classifiers. SVM and RF had better suitability with a pre-trained CNN model, which indicated these two classifiers had a broader development perspective in the hybrid bearing fault diagnosis model.

## 4. Conclusions

To evaluate different classifiers utilizing CNN feature extraction ability to improve the bearing fault diagnosis accuracy and efficiency, four hybrid models based on pre-trained CNN models and classifiers and individual classifiers were built.

In the study, firstly, the CWT method converted the bearing fault data from the CWRU dataset into CWT images and fed them into individual and hybrid models. The diagnosis accuracy and time of hybrid models and corresponding individual classifiers were compared.

From the results, (1) convolutional neural network had a distinctive improvement in bearing fault diagnosis accuracy for individual random forest (RF) in large-scale dataset and SVM model in the small-scale dataset; (2) SVM had the best performance in utilizing CNN feature extraction ability in diagnosis accuracy; (3) RF and SVM demonstrated the potential of making full use of CNN feature extraction ability in improving the diagnosis efficiency in each subclass of bearing fault.

In the further investigation, the more effective signal processing methods are supposed to be focused to reduce the effort in pre-training one CNN model to extract the fault feature. Furthermore, the deep-learning diagnosis model with high interpretability can be considered. By doing so, the process for optimizing the model can be simplified and the insights about the model mechanism have the potential to be obtained.

## Figures and Tables

**Table 1:** The details of dataset division in CWRU dataset.

Fault Type	Label	Small-Scale Dataset (Training Dataset/Test Dataset)	Large-Scale Dataset (Training Dataset/Test Dataset)
B_07	1	200/100	600/300
B_014	2	200/100	600/300
B_021	3	200/100	600/300
IR_007	4	200/100	600/300
IR_014	5	200/100	600/300
IR_021	6	200/100	600/300
OR_007	7	200/100	600/300
OR_014	8	200/100	600/300
OR_021	9	200/100	600/300
HD	10	200/100	600/300

**Table 2:** The significant hyper-parameters of classifiers in hybrid models.

Parameter	Value
Hyper-parameters of gcForest	
Input shape	28 × 28
Window size	22 × 22
Minimum samples of Multi-Grain Scanning	10
Minimum samples of Cascade Forest	2
Hyper-parameters of SVM	
Kernel	RBF
C (cost)	40
Gamma	0.0003
Hyper-parameters of RF	
Criterion parameters	Gini
N_estimators	130
Max_depth	16
Min_samples_leaf	1
Max_features	0.1
Min_samples_split	3
Hyper-parameters of LSTM	
Batch size	64
Epoch	28

**Table 3:** The experiment results.

The Average Diagnosis Accuracy Comparison of Individual Classifiers and Hybrid Models in CWRU Dataset (Percent)								
Model	RF	CNN-RF	GcForest	CNN-GcForest	SVM	CNN-SVM	LSTM	CNN-LSTM
Small-Scale Dataset	96.90	98.80	98.40	99.00	95.83	98.80	96.70	97.71
Large-Scale Dataset	95.28	98.90	98.60	99.10	97.40	99.00	98.10	98.67
**The Average Diagnosis Time Comparison of Individual Classifiers and Hybrid Models in CWRU Dataset (Second)**								
Small-Scale Dataset	0.026	0.024	0.441	0.226	0.716	0.085	3.741	1.625
Large-Scale Dataset	0.057	0.045	1.892	0.812	5.741	0.324	11.765	11.316

**Table 4:** The comparative analysis result.

The Difference between Hybrid Model Accuracy and the Corresponding Individual Classifier Accuracy (%)				
Model	RF and CNN-RF	GcForest and CNN-GcForest	SVM and CNN-SVM	LSTM and CNN-LSTM
Small-scale Dataset	1.90%	0.60%	2.97%	1.01%
Large-scale Dataset	3.62%	0.50%	1.60%	0.57%
**The ratio between diagnosis time of the hybrid model and individual classifier in the same dataset**				
Small-scale Dataset	0.923	0.512	0.119	0.434
Large-scale Dataset	0.789	0.429	0.056	0.962

## Data Availability

The data which supports the findings of this paper are available at https://engineering.case.edu/bearingdatacenter/download-data-file (accessed on 19 April 2022).
